# MetaDb: a database for metabolites and their regulation in plants with an emphasis on medicinal plants

**DOI:** 10.1186/s43897-024-00095-2

**Published:** 2024-04-29

**Authors:** Qingqing Gao, Jiajin Zhang, Juntao Cao, Chunfan Xiang, Chengxiao Yuan, Xia Li, Juan Wang, Pinhan Zhou, Lesong Li, Jia Liu, Hongchun Xie, Ruolan Li, Guilin Huang, Chaohui Li, Guanghui Zhang, Shengchao Yang, Yan Zhao

**Affiliations:** 1 Key Laboratory of Medicinal Plant Biology of Yunnan Province, National & Local Joint Engineering Research Center on Germplasms Innovation &Utilization of Chinese Medicinal Materials in Southwest China, Yunnan Agricultural University, Kunming, 650201 China; 2 Yunnan Characteristic Plant Extraction Laboratory, Kunming, 650106 Yunnan China; 3 College of Agricultural, Honghe Vocational and Technical College, Honghe, 661199 China; 4 College of Big Data, Yunnan Agricultural University, Kunming, 650201 China; 5 College of Agronomy & Biotechnology, Yunnan Agricultural University, Kunming, 650201 China

Natural product biosynthesis in medicinal plants has always been a research focus in biology. Terpenoids, phenolics, and alkaloids, all of which have medicinal value for humans, are the three most prominent natural products found in medicinal plants (Cravens et al. 2019; Li et al. 2023; Zhao et al. 2023). Many medicinal plants produce natural products that have special biological functions, such as regulating plant growth and resisting stress. Recent research has demonstrated that the biosynthesis of natural products in medicinal plants involves a complex regulatory system that integrates genes, transcription factors, and environmental factors.

Recently, multiple medicinal plant omics databases, such as An Omics Database for Herbal Medicine Plants (HMOD) (Wang et al. 2018), Applications of Integrated Multi-omics Database for Medicinal Plants (MPOD) (He et al. 2022), and An Integrated Data for Traditional Chinese Medicine Plant Genomes (TCMPG) (Meng et al. 2022), have been developed. HMOD and MPOD contain 160 genomes, 228 transcriptomes, and 5 metabolomes; TCMPG includes data for 160 medicinal plant species, 195 corresponding genomes, and 255 herbal medicines, covering almost all species of reported medicinal plants. MPOD includes the metabolic pathways for flavonoids, alkaloids, and terpenoids based on HMOD. These databases contain data on plant biosynthesis and metabolic regulation; however, they have certain limitations due to the continuous advancement of medicinal plant research.

The multidimensional data generated by genomics, transcriptomics, metabolomics, and some new omics tools have enhanced our understanding of the mechanisms by which active ingredients in medicinal plants are synthesized and regulated (Chen et al. 2023; Gao et al. 2022; Pan et al. 2015; Zhao et al. 2022). However, there is currently no updated platform for integrating plant metabolism regulation data due to the rapid iteration of data updates. Therefore, we established MetaDb (http://medmetadb.ynau.edu.cn). This database contains detailed information regarding publicly available data on medicinal plant genes, transcription factors, metabolic pathways, and metabolites, providing researchers with valuable resources for understanding the synthesis and regulation of natural products derived from medicinal plants, as well as a comprehensive resource for studying metabolic regulation in plants. Furthermore, MetaDb provides users with access to commonly used bioinformatics analysis tools, databases, and servers, including ChemDoole 2D, BLAST, and SWISS-MODEL, to help users gain a deeper understanding of the desired information.

The website homepage showcases our database, officially named MetaDb: a database for metabolites and their regulation in plants with an emphasis on medicinal plants. It provides an efficient navigation experience with different sections, including Home, Database and Tools, Browse, Server, and Help. Users can use the search box to search for gene, synthetic pathway, transcription factor, and compound data. The footer includes information for the entity maintaining the database, i.e., information about the university, laboratory, and contact address, as well as the format to cite the database. The database is deployed using the Baota panel, ensuring high availability through the Nginx reverse proxy and emphasizing data security through SSL encryption during transmission.

Browsing. The data we provide include data for genes, transcription factors, and compounds, as well as metabolic pathway maps for phenolics, terpenoids, and alkaloids from various medicinal plants. These data reflect the most recent reports. The data types are displayed in the same link, and each type includes basic information and external links. Detailed descriptions are provided for each record.

Search. The MetaDb website has been improved to include a separate search interface for users to quickly locate desired data. The search function includes two search boxes, one containing four drop-down choices and one containing an input box. Clicking the dropdown button allows users to select the type of data that can be searched in the first search box. Additionally, specific content, such as the names of genes, transcription factors, and compounds, can be entered into the input box.

Database and Tools. This section is composed of five main components: predictions, genomics, enrichments, analysis tools and chemical drawing. Each section is comprehensively equipped with a curated list of online tools and databases, enabling users to directly access resources of interest with ease. Additionally, these valuable resources and tools can be found under the " Database and Tools " menu in our navigation bar. The purpose of this section is to facilitate in-depth exploration of the systems biology of bioactive plant metabolism and regulation (Fig. 1A).

Server. Here, we aggregated three commonly used server tools: TMHMM, SWISS-MODEL, and SignalP5. Each serves a distinct purpose: TMHMM is used for the prediction of transmembrane helical structures in proteins, SWISS-MODEL is specialized for comparative protein modeling, and SignalP5 is dedicated to the online prediction of signal peptides in amino acid sequences. Our primary goal is to provide users with a comprehensive suite of analysis and prediction tools.

The homepage includes the following sections: (1) a multiselect dropdown search box; (2) a brief introduction to the database; (3) commonly used bioinformatics tool websites and database links; and (4) an entry button for phenolic, terpenoid, and alkaloid data with plant images (Fig. 1A).

**Fig. 1:**
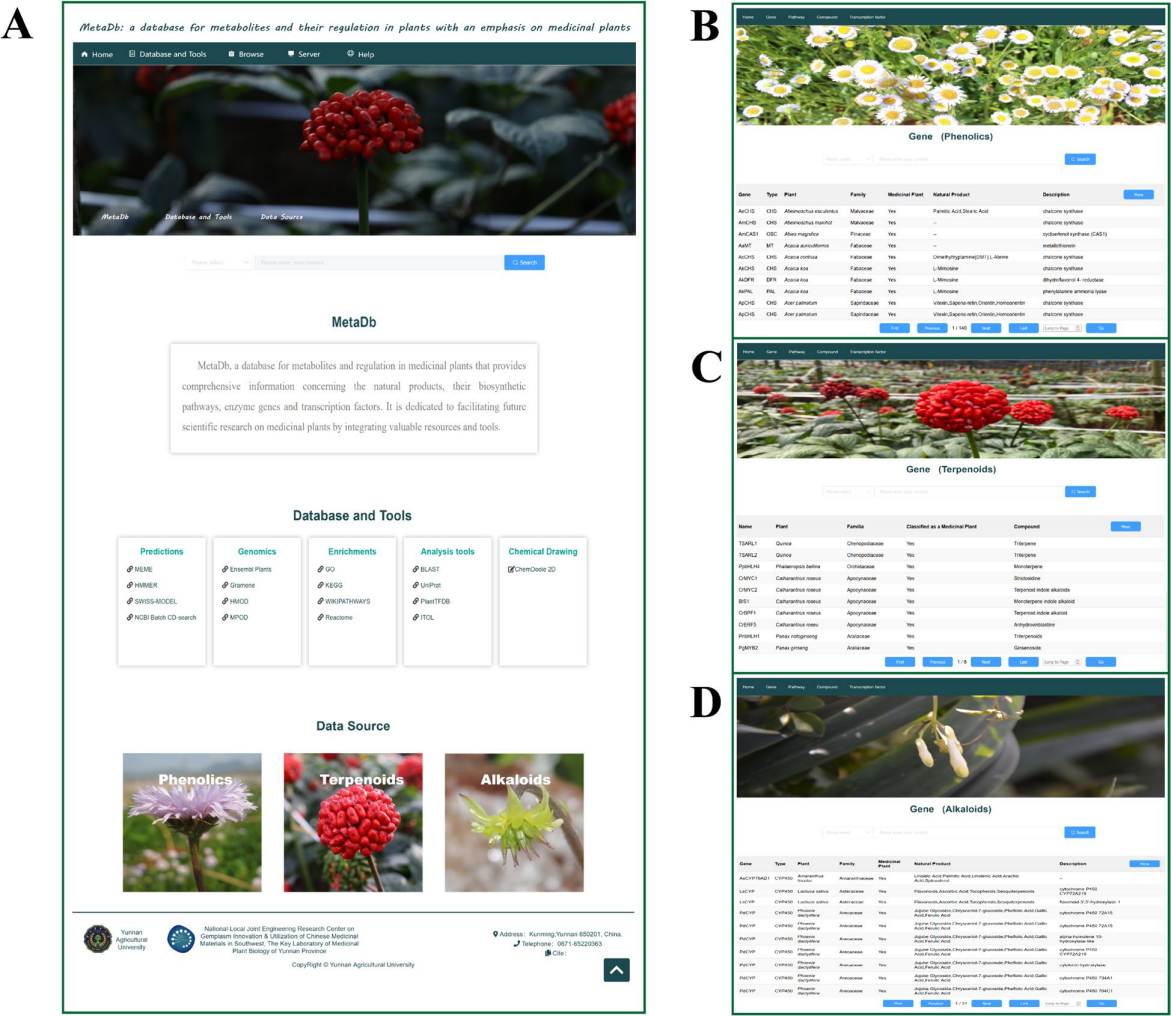
Overview of data resources and features in MetaDb. **A** The MetaDb home page. **B** Phenolic data interface for plants (Gene, Pathway, Compound and Transcription factor). **C** Terpenoid data interface for plants (Gene, Pathway, Compound and Transcription factor). **D** Alkaloid data interface for plants (Gene, Pathway, Compound and Transcription factor)

In the MetaDb, there are three main types of synthesis and regulation data for natural products from medicinal plants: phenolics, terpenoids, and alkaloids. We identified the available data through three steps. In the first step, we collected the main information and references of published medicinal plant genes, transcription factors, compounds, and metabolic pathways from MPOD (http://medicinalplants.ynau.edu.cn/), pUGTdb (https://pugtdb.biodesign.ac.cn/), and TriForC (http://bioinformatics.psb.ugent.be/triforc/). The second step was to retrieve more useful information from NCBI (https://www.ncbi.nlm.nih.gov/guide/) and TAIR (https://www.arabidopsis.org/). The third step was to classify and organize the data that was compiled and manually manage it based on characteristics such as gene type, transcription factor family classification, and compound category. If there were multiple names for genes of the same type in the same plant, all were collected.

We organized and classified the data obtained from multiple online websites and databases for future use. In MetaDb, we collected genes encoding 2,564 enzymes, 179 transcription factors, and 302 compounds from different medicinal plants. The enzyme-encoding gene data included Gene, Type, Plant, Family, Medicinal Plant, Natural Product, Description, GenBank, NCBI Link, and References. The UGT enzyme section also provides the preferred sugar donor and substrate sections, making it easier to obtain more comprehensive information when searching for the gene. The data in the transcription factor section include Name, Plant, Family, Medicinal Plant, Compound, Category, Function, and Reference. The data for the compounds include Compound, Molecular Formula, Molecular Weight, Plant, Family, Medicinal Plant, Category, Pathway link, and Reference. The compounds are associated with corresponding enzymatic reactions, whether as substrates or products.

Overall, MetaDb provides the most comprehensive information available on the biosynthesis and metabolism of natural products from medicinal plants, and it will become an important tool for promoting research on synthesis and regulation. MetaDb is a professional platform that integrates existing data to facilitate browsing and research. To ensure the availability and sustainability of MetaDb, two main measures are being taken, and we will continue to improve the database in the following areas. First, the available information from new reports is integrated into the MetaDb via manual management. Furthermore, MetaDb will be enhanced in the future to cover a broader range of information and improve the user interface. In summary, MetaDb will be continuously updated and expanded to provide useful resources on the biosynthesis and regulation of natural products in medicinal plants in the future.

## Data Availability

Not applicable.

## Abbreviations

Meta: Metabolites
Db: Database
PAL: Phenylalaninammo-nialyase
C4H: Cinnamic acid-4-hydroxylase
4CL: 4-coumarate: CoA ligase
CHS: Chalcone synthase
CHI: Chalcone isomerase
ANS: Anthocyanin synthase
F3'5'H: Flavonoid 3', 5'-hydroxylase
F3H: Flavanone 3-hydroxylase
DFR: Dihydroflavonol 4-reductase
LAR: Leucoanthocyanidin reductase
UFGT: Flavonoid 3-*O*-glucosyltransferase
FLS: Flavonol synthase
FNS: Flavone synthase
OSC: Oxidosqualene cyclase
CYP450: Cytochrome P450
UGT: UDP-glycosyltransferase
CYP450: Cytochrome P450
OMT: *O*-methyltransferase
NCBI: National Center for Biotechnology Information
CDD: Conserved domain database
GO: Gene Ontology
KEGG: Kyoto Encyclopedia of Genes and Genomes
